# Microstructure, Pitting Corrosion Resistance and Impact Toughness of Duplex Stainless Steel Underwater Dry Hyperbaric Flux-Cored Arc Welds

**DOI:** 10.3390/ma10121443

**Published:** 2017-12-18

**Authors:** Yu Hu, Yong-Hua Shi, Xiao-Qin Shen, Zhong-Min Wang

**Affiliations:** 1 School of Mechanical and Automotive Engineering, South China University of Technology, Guangzhou 510640, China; huyuscnu@163.com (Y.H.); sqshen@scut.edu.cn (X.-Q.S.); 2 Guangdong Provincial Engineering Research Center for Special Welding Technology and Equipment, South China University of Technology, Guangzhou 510640, China; 3 Guangxi Key Laboratory of Information Materials, Guilin University of Electronic Technology, Guilin 541004, China; zmwang@guet.edu.cn

**Keywords:** underwater FCAW, pitting corrosion, impact toughness, duplex stainless steel, microstructure, secondary phases, TEM

## Abstract

Duplex stainless steel multi-pass welds were made at 0.15 MPa, 0.45 MPa, and 0.75 MPa pressure, simulating underwater dry hyperbaric welding by the flux-cored arc welding (FCAW) method, with welds of normal pressure as a benchmark. The purpose of this work was to estimate the effect of ambient pressure on the microstructure, pitting corrosion resistance and impact toughness of the weld metal. The microstructure measurement revealed that the ferrite content in the weld metal made at 0.45 MPa is the lowest, followed by that of 0.75 MPa and 0.15 MPa. The analysis of potentiodynamic polarization tests at 30 °C and 50 °C demonstrated that the pitting corrosion resistance depends on the phases of the lower pitting resistance equivalent numbers (PREN), secondary austenite and ferrite. The weld metal made at 0.45 MPa had the best resistance to pitting corrosion at 30 °C and 50 °C with the highest PRENs of secondary austenite and ferrite. The weld metal made at 0.15 MPa displayed the lowest pitting corrosion resistance at 30 °C with the lowest PREN of secondary austenite, while the weld metal made at 0.75 MPa was the most seriously eroded after being tested at 50 °C for the lowest PREN of ferrite, with large cluster pits seen in ferrite at 50 °C. The impact tests displayed a typical ductile-brittle transition because of the body-centered cubic (BCC) structure of the ferrite when the test temperature was lowered. All the weld metals met the required value of 34 J at −40 °C according to the ASTM A923. The highest ferrite content corresponded to the worst impact toughness, but the highest toughness value did not correspond to the greatest austenite content. With the decreasing of the test temperature, the drop value of absorbed energy was correlated to the ferrite content. Additionally, in this work, the weld metal made at 0.45 MPa had the best combined properties of pitting resistance and impact toughness.

## 1. Introduction

Duplex stainless steels (DSSs) are characterized by an equal mixture of ferrite and austenite [1]. DSSs integrate the advantages of both phases, exhibiting a good combination of toughness, strength, as well as displaying a universal and local corrosion resistance [2]. DSSs have been an important structure material in many constructions, such as nuclear power plants, oil and gas pipelines, chemical tankers, and ocean water transportations, etc. [3,4]. Various grades of DSSs, ranging from lean to super DSSs, have been developed for different demands of mechanical and corrosion properties. For cost consideration, lean DSSs have been developed to replace standard austenite stainless steels since last century. In lean DSSs, most of the expensive Ni is substituted by Mn and N, compared with standard DSSs [5]. Mn is a stabilized element of austenite and can promote the solubility of N in lean DSS [6]. 2101 lean DSS was developed as an alternative of 304 or even 316 austenite stainless steels in late 1990s [7]. Currently, 2101 DSS is successfully used in the third generation of nuclear power plants. 

Nuclear power plants are usually located in offshore areas. Underwater welding is an important operation for the fabrication, maintenance and repair of offshore engineering [8]. Generally, underwater welding is classified as dry, local dry and wet welding according to the welding environment. The main difficulties of wet underwater welding are the negative effects of water itself. Possible effects of water include porosity, dissolved hydrogen in the weld metal, the quenching, hardening effect on the weld, and the decreased arc stability at increased water depth [9,10,11]. The quality of the DSS underwater wet welded joints are low, because the defects such as porosity caused by water cannot be avoided [12,13]. Underwater local cavity welding is restricted by the welding location and the shape of the drain cap [11,14]. Thus, to guarantee the convenience and welding quality, dry welding is a useful method without water disturbance. Some researchers have investigated the dry underwater welding technology of standard and super DSSs. Farrell [15] studied DSSs joints (Avesta 2205, Sandvik SAF2507 and Zeron 100) welded at the pressure of 0.1 Mpa and up to 3.2 MPa, and found that the hardness and impact toughness of welds at higher pressure perform better than 0.1 MPa. Akselsen [16] studied DSS 2205 joints welded with Inconel 625 metal wire at 1.2 and 3.2 MPa, and the results revealed that the two welds have similar mechanical and corrosion properties. However, the contents of alloys have obvious decreases in lean DSSs in comparison with standard and super DSSs. Chemical compositions have a big influence on the weldability and the heat input needed during welding [17]. The thermal cycle experienced during welding and the inherent chemical composition are the two major factors affecting the microstructure balance and the final performance of the welds [18]. Thus, it is necessary to study the dry underwater hyperbaric welding process and the resulting properties of lean 2101 DSS welds for the maintenance and repair of nuclear power plants.

DSSs have good weldability and usually need filler metal with higher Ni content to promote the austenite transformation from ferrite during welding [19]. Gas metal arc welding (GMAW), gas tungsten arc welding (GTAW) and flux-cored arc welding (FCAW) are the main welding methods for DSSs. GMAW or GTAW was used in most of the previous researches on dry underwater welding of DSSs [15,16,20]. However, the welding efficiency of GMAW and GTAW with solid wires is very low. Flux-cored wire can increase the welding efficiency by about 20% compared to solid wires [21]. On the other hand, the arc is concentrated and the arc force becomes stronger at higher pressure [22]. The change of arc behavior caused by increased pressure would induce the shifting of metallurgy action in the molten pool. However, studies on the effect of pressure on the metallurgy and microstructure evolution are scarce. Moreover, the balanced microstructure of dual phases is essential to good resultant properties of DSSs welds. An unbalanced microstructure is always accompanied with second precipitated phases (Cr_2_N, σ, ε, and χ etc.), which have been demonstrated to be harmful to the toughness, ductility, and the local corrosion resistance of DSS [2,4]. Thus, it is significant to reveal the effect of increasing pressure on the evolution of microstructure and properties of the joints welded using FCAW. 

The present work systematically evaluated the welding process, the evolution of microstructure, the pitting corrosion resistance, and the impact toughness of 2101 DSS FCAW weld metals in a hyperbaric chamber with pressure varying from normal pressure to 0.75 MPa. The results of this study provide useful information for improving the quality of 2101 DSS underwater welded joints.

## 2. Experimental Procedure

### 2.1. Welding Device and Materials

**Figure 1:**
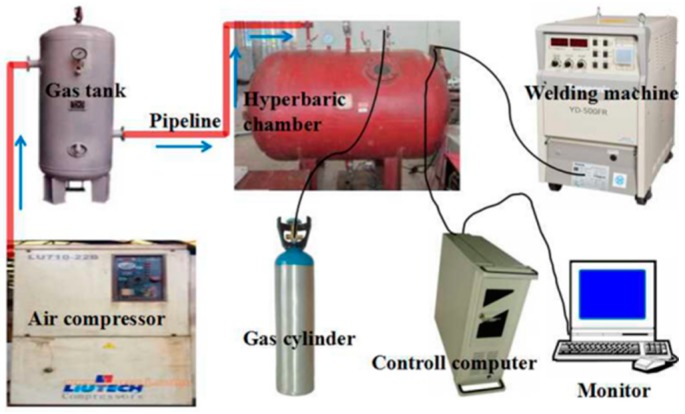
Schematic illustration of the underwater dry hyperbaric flux-cored arc welding (FCAW) system.

The underwater dry hyperbaric FCAW system is illustrated in Figure 1, mainly comprised of an air compressor, an underwater welding chamber, a PANASSONIC YD-500FR CO_2_ welding machine, a three-dimensional motion platform, a computer control center and other auxiliary equipment. The rated pressure of the hyperbaric chamber is 1.0 MPa, equivalent to a water pressure of 100 m water depth. Before welding, compressed air was pumped into the chamber by an air compressor to produce a certain ambient pressure. 

The base metal (BM) is UNS S32101 DSS. The workpieces were prepared with a dimension of 300 mm × 100 mm × 10 mm (length × width × thickness). The filler material (FM) used was LINCOLN Supercore 2205P flux-cored wire corresponding to AWS E2209-T1-1/4 class with a diameter of 1.2 mm. The chemical compositions of the BM and the FM are given in Table 1. 

### 2.2. Welding Process

**Figure 2:**
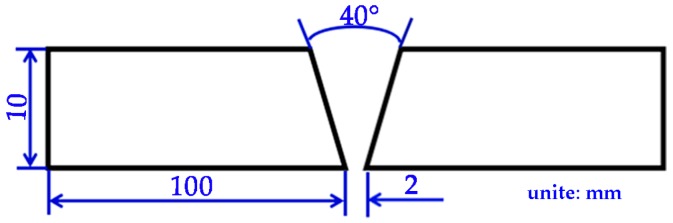
Schematic sketch of the weld joint.

The designed weld joint was a V-shape groove with a 40° groove angle, and a 2 mm root gap, as shown in Figure 2. Before welding, the plates were ground and polished until the silver, shiny metal appeared around the groove. A DCEN wire connection was used. The electrode extension was set at 15–20 mm. The shielding gas was 100% CO_2_ with a purity of 99.999% and a 20 L/min gas flow rate. The welding parameters are presented in Table 2. The ceramic backing was adopted to obtain full penetration welds with shaped back side. The slag was cleaned up after each weld bead finished and then the next weld pass was conducted. The final weld bead of Specimen 3 and Specimen 4 were conducted with the help of a welding oscillator. Three welds were prepared for each welding condition. 

### 2.3. Microstructural Examination

The machined samples were ground and polished to 2.5 μm, and subsequently etched in Beraha solution (1 g potassium metabisulphite + 30 mL hydrochloric acid + 60 mL distilled water) for 15–20 s. The average content of chemical elements in the weld metal were calculated by an Electron Probe Micro-analyzer (EPMA, SHIMADZU EPMA-1600, Kyoto, Japan) in five zones from root to surface weld with 50 times magnification. Microstructural analysis of the weld metal was made in the transverse section of the sample using optical microscopy (OM, ZEISS Axio Image M2m, Oberkochen, Germany) and scanning electron microscopy (SEM, ZEISS LEO 1530 VP, Oberkochen, Germany) combined with energy dispersive spectroscopy (EDS, Oxford X-MaxN20, Taunusstein, Germany). The EDS measurements were repeated at least ten times. The contents of ferrite were counted with the Image-Pro Plus software. The OM pictures of each specimen were captured and processed to calculate the average content of ferrite at a magnification of 100 times. Transmission electron microscopy (TEM, TECNAI F20, Hillsboro, OR, USA) was used to further identify the tiny precipitates. 

### 2.4. Electrochemical Measurement and Impact Toughness Test

**Figure 3:**
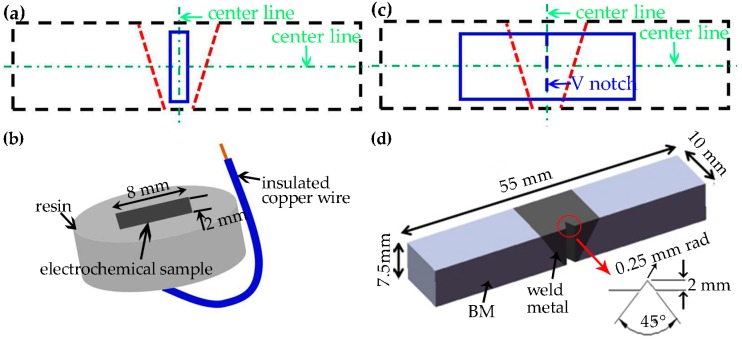
Schematic diagrams of the electrochemical test sample and the impact toughness sample: (**a**,**b**) sampling location and package illustration of the electrochemical test specimen; (**c**,**d**) sampling location and stereo view of the impact toughness specimen. (The rectangles with blue solid lines represent the tested samples in (**a**,**c**)).

The specimens for the electrochemical measurements were mechanically ground using silicon carbide paper and were finished by polishing with a 2.5 μm diamond paste. The exposed area of the tested sample was 0.16 mm^2^. The sampling location and the illustration of the specimen for potentiodynamic polarization test are shown in Figure 3a,b. All the electrochemical measurements were carried out in 1 mol/L NaCl solution using a three-electrode potentiostat (CHI600E). A platinum sheet was used as the counter electrode, a standard saturated calomel electrode (SCE) was used as a reference electrode and the specimen was used as the working electrode. The test solutions were made up of analytical grade reagent and distilled water. Before the potentiodynamic polarization test, an open-circuit potential (E_ocp_) test was executed for 20 min. Potentiodynamic polarization curves were gained respectively at 30 °C and 50 °C with a scan rate of 1 mv/s starting from E_ocp_-300 mV_SCE_ to the potential where current density exceeded 10^−2^ A/cm^2^. The solution temperature was held constant using a water bath. In addition, each test was reproduced three times to ensure reliability.

Figure 3c shows the sampling schematic diagrams and Figure 3d presents the sub-size Charpy V-notch samples for impact toughness with 55 mm × 10 mm × 7.5 mm according to ASTM A370 standard [23]. Charpy V-notch impact tests were achieved at 25 °C, −40 °C, −80 °C, and each test was repeated three times. 

## 3. Results and Discussion

### 3.1. Thermodynamic Analysis

**Figure 4:**
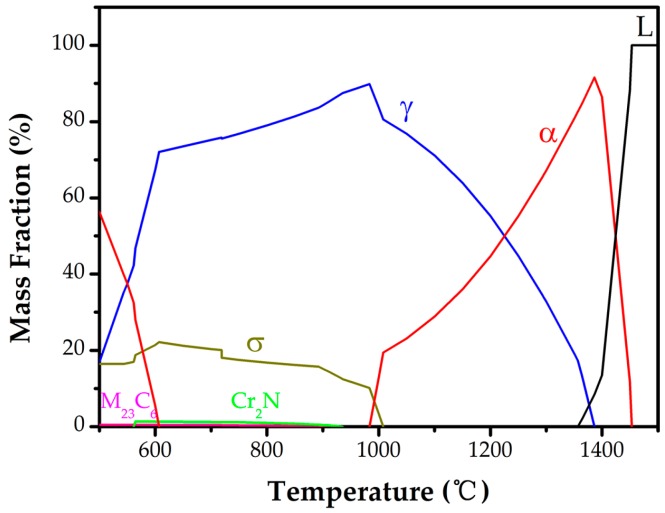
The equilibrium phase transition diagram.

**Figure 5:**
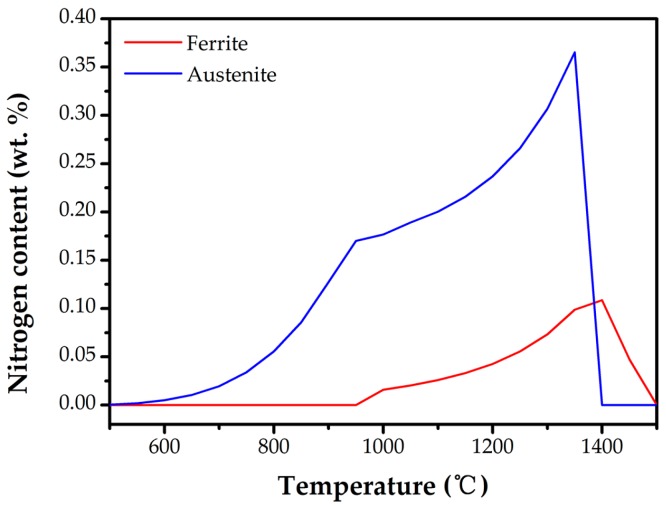
The nitrogen solubility vs. temperature in ferrite and austenite.

To illustrate the existing phases and microstructures in the weld metal, the equilibrium phase transition diagram was calculated with the help of JMatPro software, as shown in Figure 4. The plots exhibit a balanced transformation procedure: Liquid (L) → L + ferrite (α) → L + α + austenite (γ) → α + γ + second precipitates, and the second precipitates include σ, M_23_C_6_, and Cr_2_N. Namely, there is a minor co-existence temperature range for ferrite and austenite during solidification. In the next solid-state phase transition, ferrite partially transforms to austenite within a large temperature range when the temperature falls to the γ solvus temperature. Moreover, the temperature dependent nitrogen solubility in each single phase was calculated in Figure 5. The nitrogen solubility in austenites is much greater than that in ferrites. Usually there is only about 0.05% saturation in ferrites), as the solubility of N is higher in face centered cubic (FCC) austenite than in body-centered cubic (BCC) ferrite [24]. However, the ferrite does not have enough time to transform to austenite during welding for the rapid cooling. As a result, the inadequate austenite transformation induces supersaturated nitrogen in the ferrite phase and chromium nitride precipitated in the ferrite matrix. During multi-pass welding, the meta-stable phase provides nucleation sites for secondary austenite γ_2_, and would be replaced partially or fully by γ_2_. In this study, no other second precipitates were observed.

### 3.2. Microstructures Characterization

**Figure 6:**
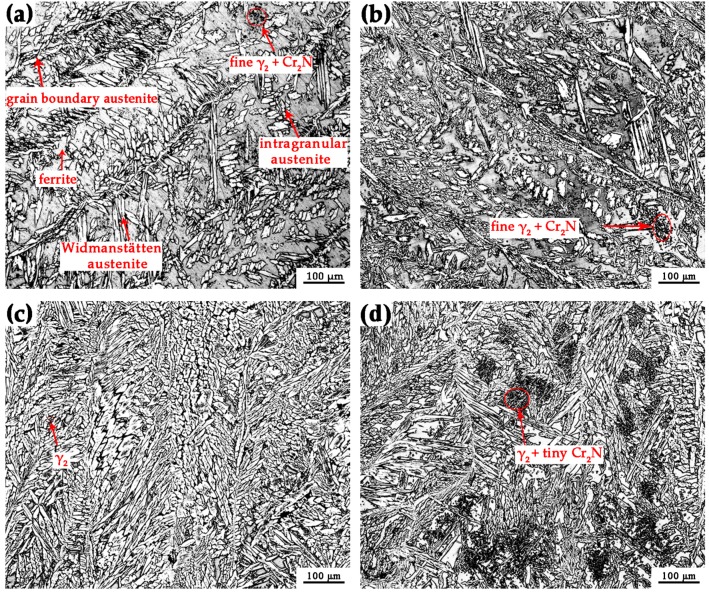
Optical microstructures of the central weld metals: (**a**) Specimen 1; (**b**) Specimen 2; (**c**) Specimen 3; (**d**) Specimen 4.

**Figure 7:**
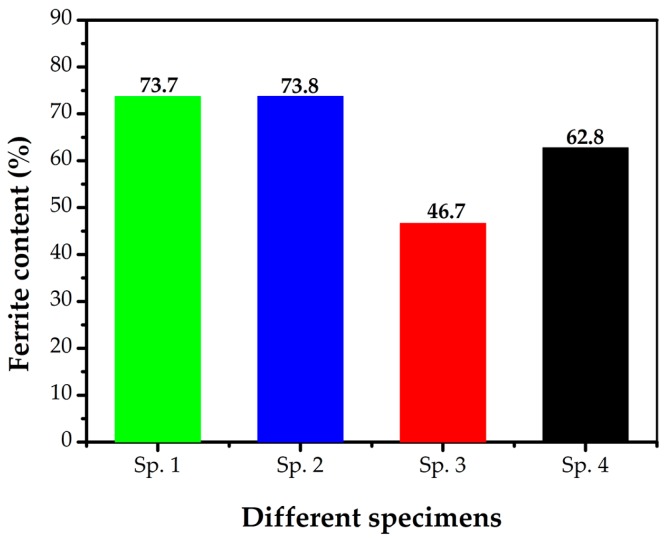
Ferrite contents of different specimens.

All the welds had no welding defects as indicated by X-ray nondestructive imaging. From Table 2 we can conclude that it needs more weld passes to fill the groove and a smaller heat input for the backing weld when the pressure is elevated. When welding in a hyperbaric environment, the arc column gets shorted and the arc diameter is decreased using a constant welding power, and as a result, the arc energy is concentrated and the arc force is stronger [25]. That is why the higher the ambient pressure, the lower the root heat input, when using the same weld joint design, as shown in Table 2. That then leads to the increase in the numbers of weld passes to fill the weld joint. Figure 6 shows the optical microstructures of filler metals welded at different ambient pressures. Based on the phase transformation mechanism during multi-pass welding, the austenites included primary austenite γ_1_ and secondary austenite γ_2_. Most of the primary austenites came from the transformation of ferrites and a little from the liquid metal. With the decrease in temperature, the primary austenites morphology successively emerged as grain boundary amorphous structures, Widmanstätten side plates or intragranular side plates, acicular and fine intragranular precipitates [26]. When the microstructure was reheated during the next welding pass between 800 and 1200 °C, additional austenites appeared, which were secondary austenites with much smaller sizes than the primary austenites [21]. We also measured the average main alloy element contents of each phase (ferrite, primary austenite, secondary austenite) using EDS analyzer, as shown in Table 3. Based on the previous studies on chemical composition of ferrite and austenite, it was found that Cr and Mo were enriched in ferrite whereas there was more Ni content in austenite in all the specimens [24,27]. The data in Table 3 also suggested lower element content of Cr, Mo in γ_2_ than primary austenite. With the increase in ambient pressure from 0 to 0.75 MPa, there was an significant variation of the ferrite content in the weld metals when the ambient pressure is within the range of 0.15 to 0.75 MPa, while the ferrite content is almost the same for the weld metals welded at atmospheric pressure and 0.15 MPa ambient pressure, as shown in Figure 7. The reasons for the differences in ferrite content of the welding metals at different welding pressures will be discussed in Section 3.3.

**Figure 8:**
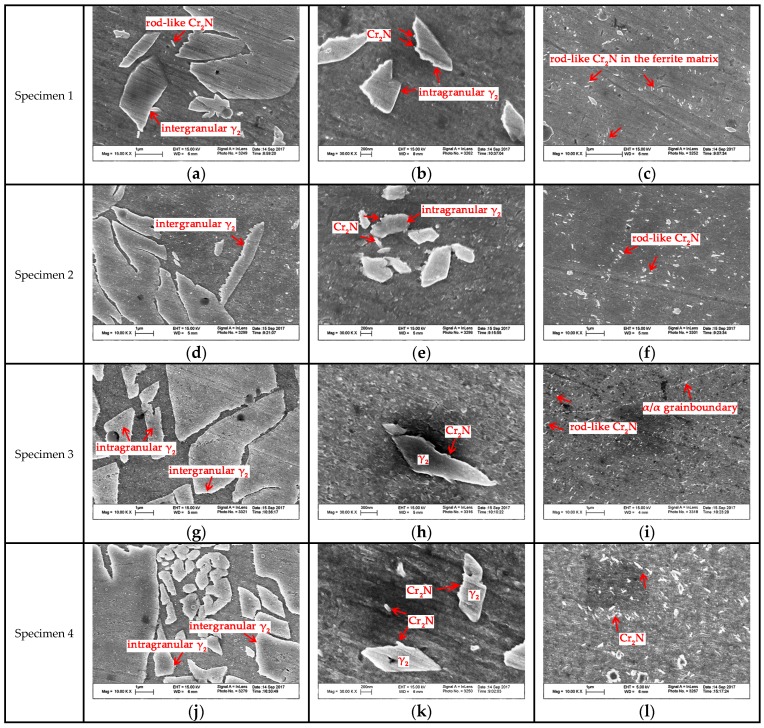
SEM graphs of γ_2_ and Cr_2_N in each specimen: (**a**–**c**) of Specimen1; (**d**–**f**) of Specimen 2; (**g**–**i**) of Specimen 3 and (**j**–**l**) of Specimen 4.

**Figure 9:**
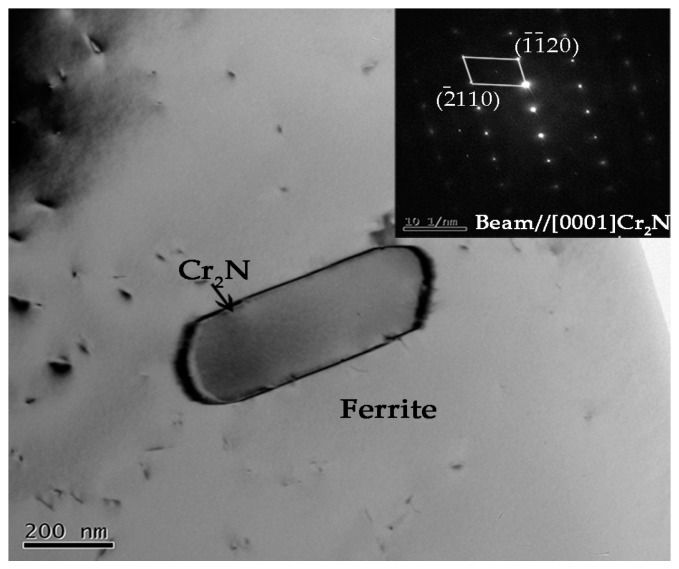
General bright field TEM micrograph of rod-like chromium nitride precipitated in ferrite matrix and the corresponding diffraction patterns.

The magnified SEM photos of secondary austenite γ_2_ and chromium nitrides are shown in Figure 8. There were two types of γ_2_ reported: Intergranular γ_2_ formed at α/γ_1_ interface and intragranular γ_2_ nucleated in the ferrite matrix [28]. The intergranular γ_2_ is the continued growth of primary austenite as a direct homogeneous nucleation at γ_1_, while the intragranular γ_2_ nucleates at intragranular inclusions, dislocations or precipitates and grows controlled by diffusion [28]. The TEM graph and corresponding diffraction pattern of the nitrides revealed the rod-like chromium nitride Cr_2_N, as shown in Figure 9. Either chromium nitride or γ_2_ suppresses the corrosion resistance of DSSs, but γ_2_ can improve the toughness of weld joints while chromium nitrides are harmful to the toughness [27,29,30,31,32].

### 3.3. Effect of Ambient Pressure on the Microstructure

**Figure 10:**
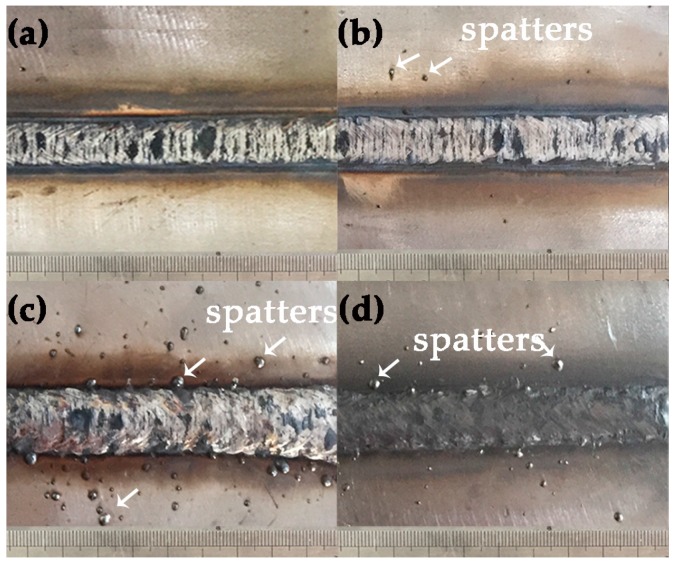
Appearance of welds (**a**) Specimen 1; (**b**) Specimen 2; (**c**) Specimen 3; and (**d**) Specimen 4.

The influence of ambient pressure on the welding process during dry hyperbaric welding could be concluded as follows: (і) the cooling rate increases with the increase in pressure during underwater hyperbaric welding. The thermal conductivity of the pressurized gas in the chamber increases with the increasing of the pressure, due to higher gas density of increased pressure [33]. (ii) The arc instability increases with the increase in pressure. It is generally understood that, more welding power is needed to hold the arc at a higher pressure, but the extra energy is not transferred to the molten pool [34]. In this work, the values of welding voltage and current used for all the welding processes were set up in accordance with those at normal pressure. Incongruous welding power with increased pressure could result in spattering, especially for 0.45 MPa weld metal as shown in Figure 10. (iii) The instability of shielding gas flow occurs at an interval of 5–8 bar pressure (equivalent to 0.5–0.8 MPa) in the form of turbulent flow [15]. As presented in Figure 7, the weld metals of Specimen 1 and Specimen 2 had nearly equal ferrite content, which were higher than Specimen 3 and Specimen 4, and Specimen 3 had the best balanced microstructure. 

The chemical composition of the weld metals are shown in Table 4. The significant difference in Table 4 is the extraordinarily high content of N (0.27%) in Specimen 3. The N element is more important to the austenite formation than the substitutional solute elements (Mn, Ni) since it can be better diffused when encountering the rapid cooling during welding [35,36], which is an additional reason that austenite content is superior to the ferrite content. However, it is well known that higher heat input and slower cooling rate can promote more ferrite transformation to austenite during welding [32,37,38]. Recalling Table 2, the total heat inputs of Specimen 3 (3160 kJ/mm) were more than Specimen 4 (2966 kJ/mm), also Specimen 1 and Specimen 2 (both 2376 kJ/mm), which is another reason there are more austenite contents in Sp. 3 than the other three specimens. 

### 3.4. Electrochemical Test

**Figure 11:**
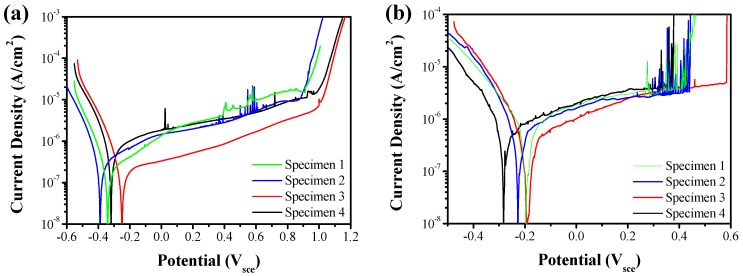
The polarization curves of fusion zones of the as-weld specimens: (**a**) 30 °C; (**b**) 50 °C.

**Figure 12:**
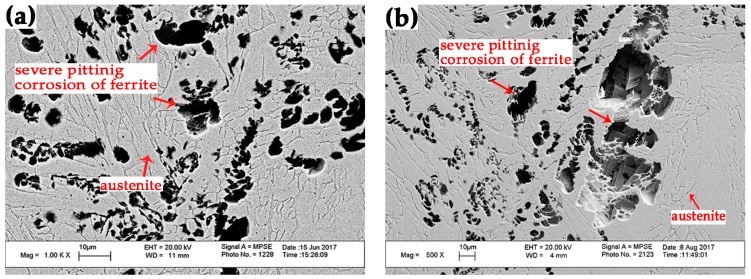

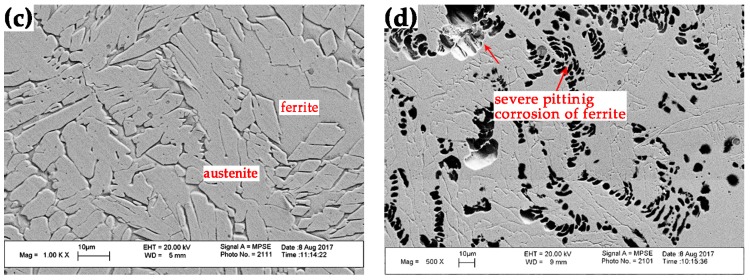
SEM images of pitting morphologies formed at 0.5 V_sce_ at 50 °C: severely pitting corrosion in ferrite of (**a**) Specimen 1, (**b**) Specimen 2 and (**d**) Specimen 4; and no obvious cluster pits of (**c**) Specimen 3.

**Figure 13:**
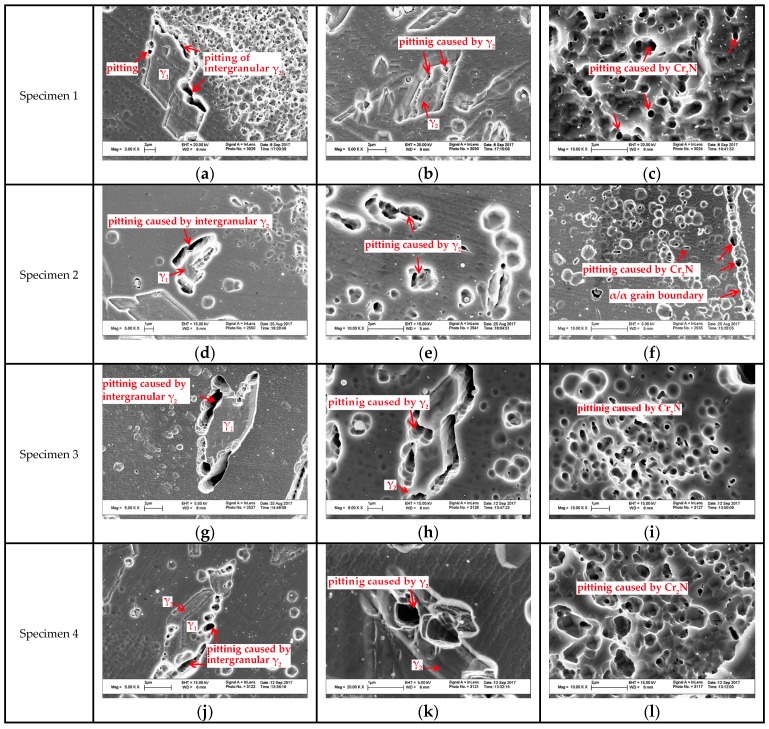
SEM images of pitting morphologies of each specimen after electrochemical polarization test: (**a**–**c**) of Specimen 1; (**d**–**f**) of Specimen 2; (**g**–**i**) of Specimen 3 and (**j**–**l**) of Specimen 4.

To study the pitting corrosion resistance evolution of weld metals obtained at each ambient pressure, as mentioned above, the potentiodynamic polarization curves were conducted at 30 °C and 50 °C. The classical curves are shown in Figure 11, and all the curves did not reveal an apparently obvious active to passivation range. The potential at which the current density reached 10^−4^ A/cm^2^ was taken as the pitting potential. The pitting potential *E*_pit_ was used for estimating the corrosion resistance of the weld metal, and typically a better corrosion resistance would be associated with a higher pitting potential *E*_pit_ value. The pitting potentials *E*_pit_ of the four curves at 30 °C were presented in descending order as Specimen 3, Specimen 4, Specimen 2 and Specimen 1 (1092, 1073, 956 and 987 mv), however, those of 50 °C were changed as Specimen 3, Specimen 2, Specimen 1 and Specimen 4 (586, 444, 421 and 379 mv). The pitting potentials of Specimen 1 and Specimen 2 were nearly the same, which told us that Specimen 1 and Specimen 2 have similar pitting resistance. When the temperature increased from 30 °C to 50 °C, the pitting potentials decreased by nearly 50%–60%, especially in Specimen 4 which went from 987 mv to 379 mv. This depleted pitting corrosion resistance resulted from the accelerated dissolution of passive film at higher temperature, which is in accordance with the result in the study of Kang [39]. When examining the pitting morphology of samples tested at 30 °C, there was no stable pits cluster observed using SEM. However, the samples of Specimen 1, Specimen 2 and Specimen 4 tested at 50 °C were seriously eroded with apparent pitting holes spreading in the ferrite, while there were still no stable pits observed in Specimen 3, as shown in Figure 12. Moreover, when tested with larger magnification, metastable pits could be seen in all the samples, scattered at α/γ_2_ boundaries, within γ_2_, or around to strip the Cr_2_N particles within α matrix, as demonstrated in Figure 13. Similar localized corrosion morphologies were also observed by other researcher [2,40,41].

The pitting resistance equivalent number (PREN) was known to evaluate the pitting corrosion resistance. Usually a larger PREN reflects superior pitting resistance [24]. To further study the variation of the pitting resistance of the specimens, the average PREN of ferrite, primary austenite and secondary austenite are listed in Table 3. The PREN was calculated according to the classical expression [41] as follows:

PREN = %Cr + 3.3%Mo + 16%N(1)

Comparing the PRENs of phases in all the specimens, the PRENs of ferrite were all lower than those of primary austenites, but higher than those of secondary austenites. Namely, the secondary austenite is the weakest phase and thus the first to be eroded. This is in agreement with the observation of pitting corrosion morphology in Figure 13. Previous studies also found that secondary austenite and ferrite are easier to be corroded than other phases during the pitting tests [27,42]. Because the PREN values of secondary austenite in the four specimens were in order as 30.849, 30.693, 32.931 and 31.886 for Specimen 1–4, the pitting corrosion resistance could be ranked as Specimen 3, Specimen 4, Specimen 1 and Specimen 2. The pitting corrosion began at the secondary austenite, but the percentage of secondary austenite was much less in comparison to the main phases of austenite and ferrite. Then the pitting must expand to the inferior phase of ferrite. Moreover, the eroded secondary austenites in these specimens were located in the ferrite matrix, which caused the pitting propagation to continue with less hindrance. Once cluster stable pits occurred in ferrite, the PREN of ferrite determined the pitting corrosion resistance of the sample [42]. Considering that the PREN values of ferrites in Specimen 1 and Specimen 2 were slightly higher than that in Specimen 4 (32.491, 32.418 > 32.305), the pitting resistances of Specimen 1 and Specimen 2 were slightly better than Specimen 4, though stable and severe cluster pits of ferrite were seen in the three specimens at 50 °C. For Specimen 3, the PREN value of ferrite was up to 34.015 and no big pits were seen in the ferrite of Specimen 3. However, the superior corrosion resistance of Specimen 3 could also be attributed to the better-balanced two-phase microstructure compared with the other three weld metals. The excellent corrosion resistance of DSS is the result of the balanced two phases and that is what causes the development of DSS steels [39]. Once the balance is broken, the fine properties will disappear. Austenite is just like a barrier to defend external invasions [38]. Additionally, the precipitation of chromium nitrides was critical to the degradation of pitting corrosion resistance [43,44]. As these Cr_2_N precipitates are concentrated in Cr, N with a little Fe, Ni, and Mo, the Cr depleted region around Cr_2_N would be unable to prevent the pitting initiation [21]. Thus, the existence of Cr_2_N in the ferrite during welding is an accomplice to the deterioration caused by pitting corrosion. 

### 3.5. Impact Toughness

**Figure 14:**
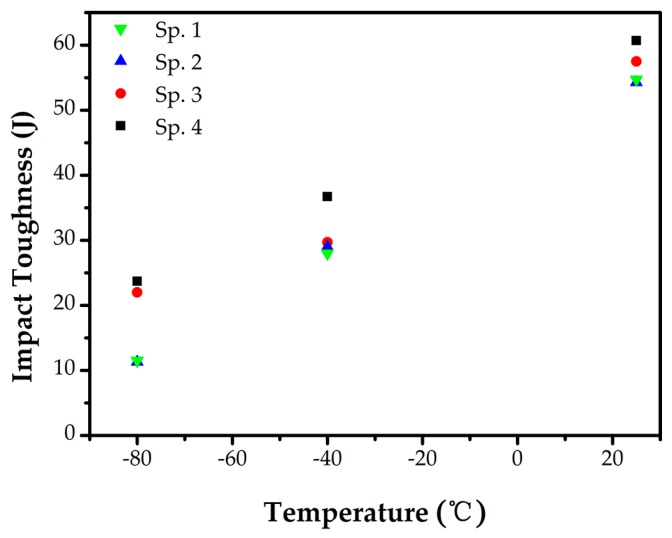
Charpy-V impact toughness of weld metals welded at different ambient pressure.

The impact energy evaluated by Charpy-V notch tests of the dry hyperbaric FCAW butt weld metals at 25 °C, −40 °C, and −80 °C are presented in Figure 14. The impact toughness of all the three weld metals decreased with test temperature lowering. The result showed that the weld metal of Specimen 3 has the highest absorbed energy, while those of Specimen 1 and Specimen 2 have the least absorbed energy. All the specimens tested at −40 °C could meet the minimum impact energy requirement of 25.5 J according to ASTM A923 (reduced in direct proportion relative to full size specimen of 34 J). The weld metals of Specimen 1, Specimen 2 and Specimen 3 displayed almost the same impact toughness at −40 °C, but the ones of Specimen 1 and Specimen 2 both exhibited a more distinct fall at −80 °C. However, the difference of the impact toughness of the weld metals of Specimen 3 and Specimen 4 became smaller at a lower test temperature. Previous works have already discovered that ferrite content ranging from 10 FN to 60 FN has no corresponding relation to the impact toughness value for duplex weld metals [45]. However, Kang illustrated a discordant phenomenon of a reduction in absorbed energy along with increased ferrite content from 42 FN to 61 FN [46]. However in this examination, the toughness values of the three weld metals showed that the lowest austenite content is in agreement with the worst impact toughness, whereas the highest austenite content does not correspond to the best impact toughness. The absorbed energy of weld metal of Specimen 3 with the highest austenite content was lower than that of Specimen 4. That suggested the austenite content was not the critical, decisive factor for the impact toughness. 

**Figure 15:**
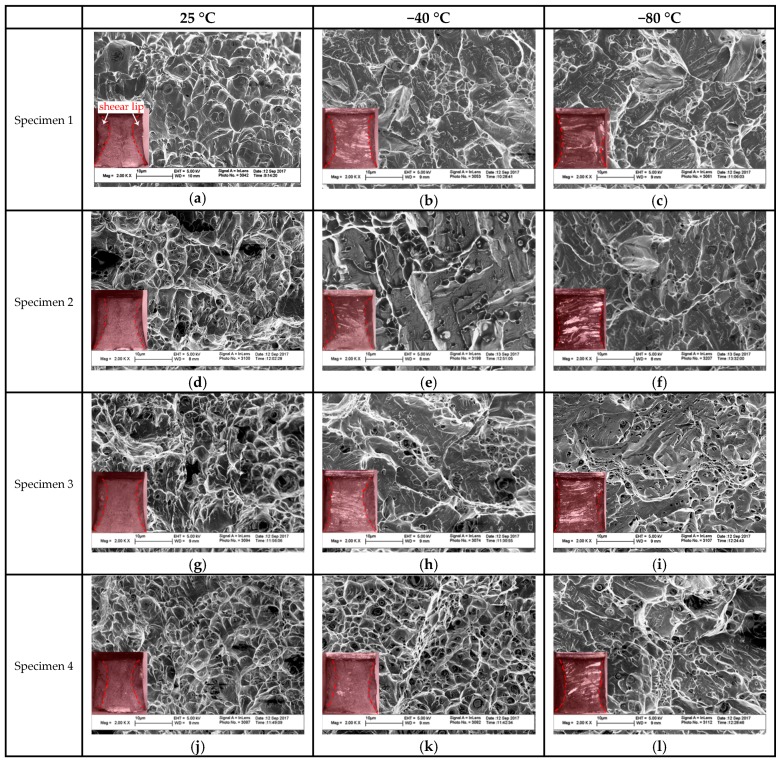
SEM fracture surfaces of weld metals after impact test: (**a**–**c**) of Specimen 1; (**d**–**f**) of Specimen 2; (**g**–**i**) of Specimen 3 and (**j**–**l**) of Specimen 4.

**Figure 16:**
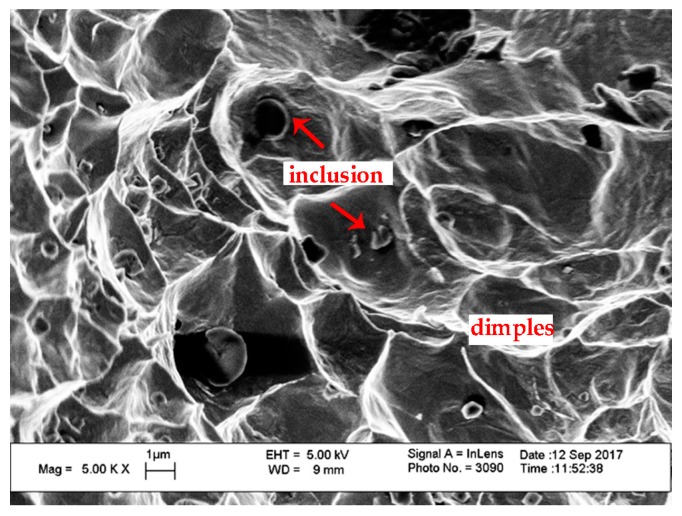
Inclusions in the dimples.

**Figure 17:**
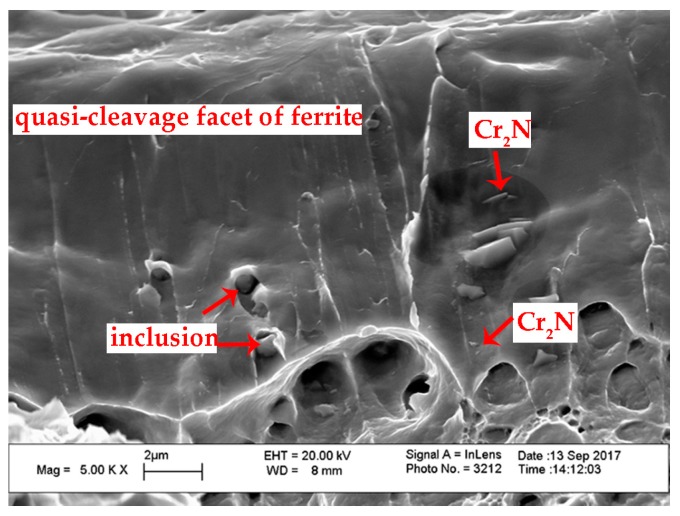
Chromium nitrides observed in the impact fracture surface.

Figure 15 shows the fracture micrographs of weld metals obtained at different pressures. From Figure 15 we can see that all the weld metals display ductile fractures at 25 °C, with small, dense and isometric dimples. The dimples nucleated at the inclusions and grew, as displayed in Figure 16. The three specimens tested at −40 °C experienced a ductile-brittle mixed fracture, as the fracture surfaces were all comprised of varied, relatively small degrees of brittle fracture, which were revealed by the showing up of quasi-cleavage facets. More than half of the fracture surface were occupied by the bright facet at −80 °C, which indicated a brittle fracture mechanism. The existing, so-called dimples, were stretched out of shape and then became shallow or bursting clusters with tearing edges when tested at −80 °C of Specimen 3 and Specimen 4. The weld metals of Specimen 1 and Specimen 2 fractured at −80 °C showed almost full brittle fractures with no significant shear lips at the fracture edges of the macroscopic fracture morphologies. Analyzing the chemical content of the cleavage crystal facets and the dimple regions of one weld metal of Specimen 2 using EDS, the result showed that the cleavage facets have a higher Cr content (20.56%) and lower Ni content (5.87%) than that of dimple regions (19.31%, 5.98%). This again confirmed that the cleavage fracture generated in the ferrite phase. High definition pictures at higher magnification showed Cr_2_N precipitates in the quasi-cleavage facets in Figure 17, which suggested that Cr_2_N caused the decrease in absorbed impact energy of the weld metals. There were occasionally arising dimples scattered in the facet, according to Kang, these torn shallow dimples resulted from the fracture of secondary austenite [46].

Ferrite is the inferior ductile phase of the two phases, with a body-centered cubic (BCC) lattice structure. Moreover, Karlsson found that in a primary ferrite mode, the ferrite morphology is beneficial to continuous crack propagation and that is why higher ferrite content would be anticipated with a lower impact toughness [45]. Furthermore, the excellent impact property of DSS is attributed to the balanced phase content. Usually, high ferrite content goes along with Cr_2_N precipitates and many studies have demonstrated that the Cr_2_N precipitates would severely impair the impact toughness of DSS [46,47]. Also, in Wang’s opinion [48], the almost equal amounts of the two phases can create more uniform the bi-phase grain size, smooth phase boundaries, and suppress the crack propagation. These opinions explain the worst toughness of 0 and 0.15 MPa, but they cannot rationalize the absorbed energy of weld metals welded at 0.45 MPa with the highest austenite content. In the work of Pilhagen, the weld metals of 6 Ni had a nearly double impact toughness value of that of 5 Ni weld metals, though there were similar microstructure morphology and ferrite content [49]. Also Li [50] found that the addition of Ni can reduce the fracturing of the ferrite phase. Regarding this view, the impact toughness of 0.45 MPa weld metal was not as good as expected for the highest austenite content, and the reason can be attributed to the Ni content loss, recalling Table 3.

As to the subzero impact toughness, as we all know, the ferrite phase has a ductile-brittle characteristic. The preferential growth orientation [100] of ferrite is in conjunction with the easiest cleavage planes {100}, which would seriously damage the resulting toughness below the ductile to brittle transition temperature (DBTT) [45,51]. According to He [52], the density of dislocations existing at the mechanical twin boundaries or in the twins of ferrite above DBTT was much lower than that below the DBTT, and the fracture of twin controlled the brittle fracture at low temperatures. To summarize, the higher the ferrite content, the higher the toughness gap when specimens are tested below the DBTT. Thus, it is easy to understand that the absorbed energy of 0.45 MPa weld metal with the highest austenite content reduced least when tested from 25 °C to −80 °C.

## 4. Conclusions

The underwater dry hyperbaric FCAW multi-pass butt joints of S32101 DSS plates were conducted in a chamber at the pressures of 0–0.75 MPa. Comparing the microstructure, pitting corrosion resistance and impact toughness of the weld metals, the findings can be outlined as follows:

(1) The heat input for root weld should decrease with the increase in ambient pressure to obtain a uniform weld shape, and consequently the number of weld layers to fulfill the groove’s needs to be increased, compared to welding at lower pressures with the same groove types.
(2) The weld metal made at 0.45 MPa had the most content of austenite followed by 0.75 MPa, and 0.15 MPa. The specimen obtained at 0.15 MPa behaved almost the same as the specimen of normal pressure. The most significant difference in the chemical composition is the much higher concentration of N in the weld metal made at 0.45 MPa than the other three specimens. The more the austenite content, the less presence of the chromium nitrides in ferrite.
(3) The phases of lower PRENs reflected the pitting corrosion resistance of the weld metal measured by potentiodynamic polarization test in 1 mol/L NaCl solution at 30 °C and 50 °C. The metastable pits appearing at the α/γ_2_ boundaries, γ_2_ and around the Cr_2_N were viewed in all the specimens tested at the two test temperatures. Stable and clustered pits were not seen in specimens tested at 30 °C, while severely eroded pitting holes could be observed in ferrite of 0, 0.15 and 0.75 MPa tested at 50 °C. The weld metal made at 0.75 MPa had better pitting resistance than those made at 0 and 0.15 MPa at 30 °C because of higher PREN of secondary austenite of the 0.75 MPa weld metal, while the situation was reversed at 50 °C because the PREN of ferrite of the 0.75 MPa weld metal is smaller. Therefore, the weld metal made at 0.75 MPa experienced bad pitting resistance. The weld metal of 0.45 MPa presented the best resistance to pitting propagation in all the tests.
(4) At the three tested temperatures (25 °C, −40 °C, and −80 °C), the impact toughness had no direct relation to the austenite content. The weld metal made at 0.75 MPa expressed the highest toughness value in comparison with the other weld metals and all the absorbed energy had a decline in different degrees along with a ductile-brittle transition. Whereas, the loss of toughness values were inversely proportional to the percentage of ferrite.
(5) The ridiculously high ferrite content in the weld metals as well as the Cr_2_N precipitates had a serious effect on the pitting corrosion resistance and the absorbed energy. In addition, the weld metal made at 0.45 MPa had the best comprehensive performance of pitting corrosion resistance and impact toughness in this study.

## Figures and Tables

**Table 1:** Chemical composition of base metal (BM) and filler metal (FM) (wt %).

	C	Si	Mn	Cr	Ni	Mo	Cu	Co.	P	S	N	PREN
**BM**	0.017	0.49	4.98	21.52	1.56	0.22	0.16	0.04	0.02	<0.002	0.24	26.1
**FM**	0.024	0.63	1.57	22.07	8.17	3.05	0.019	-	0.021	0.009	0.15	34.54

**Table 2:** Welding parameters used in underwater dry hyperbaric multi-pass FCAW.

Specimen	Gauge Pressure (MPa)	No. of Layers	Current (A)	Voltage (V)	Welding Speed (mm·s^−1^)	Heat Input (J·mm^−1^)
Specimen 1	0 (normal atmosphere)	1 (root)	170	28	4	1190
		2 (finishing)	180	29	4.4	1186
Specimen 2	0.15	1 (root)	170	28	4	1190
		2 (finishing)	180	29	4.4	1186
Specimen 3	0.45	1 (root)	160	27	4	1080
		2 (filling)	170	28	4.8	992
		3 (finishing)	180	29	4.8	1088
Specimen 4	0.75	1 (root)	150	26	4.4	886
		2 (filling)	170	28	4.8	992
		3 (finishing)	180	29	4.8	1088

**Table 3:** Main alloy elements contents of each phase (wt %).

Specimen	Phase	Cr	Ni	Mo	N	PREN
Specimen 1	ferrite	23.37	6.74	2.57	0.04	32.491
	primary austenite	22.86	7.13	2.37	0.28	35.161
	secondary austenite	21.77	8.19	1.83	0.19	30.849
Specimen 2	ferrite	23.33	6.84	2.56	0.04	32.418
	primary austenite	22.79	7.36	2.43	0.27	35.129
	secondary austenite	21.36	8.34	1.81	0.21	30.693
Specimen 3	ferrite	23.47	6.79	3.05	0.03	34.015
	primary austenite	22.99	7.54	2.52	0.23	34.986
	secondary austenite	22.41	8.02	2.17	0.21	32.931
Specimen 4	ferrite	23.41	6.49	2.55	0.03	32.305
	primary austenite	22.95	6.89	2.32	0.23	34.286
	secondary austenite	22.03	7.22	1.92	0.22	31.886

**Table 4:** EPMA analysis of the weld metals showing the average value (wt %). Min–max value in parentheses. Nitrogen content measured by infrared combustion method.

Specimen	Si	Mo	Cr	Mn	Fe	Ni	N
Specimen 1	0.558	2.148	21.872	2.177	65.7	6.848	0.16
	(0.55–0.57)	(1.91–2.64)	(21.67–22.07)	(2–2.56)	(64.95–66.56)	(6.43–7.39)	
Specimen 2	0.556	2.164	21.708	2.164	65.656	6.77	0.17
	(0.47–0.61)	(1.90–2.49)	(21.55–22.06)	(1.84–2.47)	(64.68–66.31)	(6.32–7.37)	
Specimen 3	0.612	2.226	21.824	2.182	65.198	6.856	0.27
	(0.56–0.65)	(0.00–2.71)	(21.44–22.23)	(1.55–2.51)	(63.51–65.74)	(6.25–8.09)	
Specimen 4	0.554	2.298	21.7	1.93	65.14	7.378	0.16
	(0.48–0.66)	(2.07–2.57)	(21.53–22.00)	(1.51–2.4)	(64.5–65.46)	(6.67–8.36)	
